# Ultrasound Features and Clinical Outcome of Patients with Ovarian Masses Diagnosed during Pregnancy: Experience of Single Gynecological Ultrasound Center

**DOI:** 10.3390/diagnostics13203247

**Published:** 2023-10-18

**Authors:** Matteo Bruno, Giulia Capanna, Veronica Stanislao, Raffaella Ciuffreda, Sara Tabacco, Ilaria Fantasia, Christian Di Florio, Guglielmo Stabile, Angela D’Alfonso, Maurizio Guido, Manuela Ludovisi

**Affiliations:** 1 Department of Obstetrics and Gynecology, San Salvatore Hospital, 67100 L’Aquila, Italy; brunomatteo2@gmail.com (M.B.); saratabacco87@gmail.com (S.T.); ilariafantasia@gmail.com (I.F.); christiandiflo@gmail.com (C.D.F.); 2 Department of Clinical Medicine Life Health and Environmental Sciences, University of L’Aquila, 67100 L’Aquila, Italy; veronicastanislao@gmail.com (V.S.); raffaella.ciuffreda@student.univaq.it (R.C.); angela.dalfonso@univaq.it (A.D.); maurizioguido@libero.it (M.G.); mludovisi@gmail.com (M.L.); 3 Department of Obstetrics and Gynecology, Institute for Maternal and Child Health—IRCCS “Burlo Garofolo”, 34137 Trieste, Italy; guglielmost@gmail.com

**Keywords:** adnexal masses, pregnancy, ultrasound, experienced sonographer

## Abstract

(1) Background: The number of adnexal masses detected during pregnancy has increased due to the use of first-trimester screening and increasingly advanced maternal age. Despite their low risk of malignancy, other risks associated with these masses include torsion, rupture and labor obstruction. Correct diagnosis and management are needed to guarantee both maternal and fetal safety. Adnexal masses may be troublesome to classify during pregnancy due to the increased volume of the uterus and pregnancy-related hormonal changes. Management should be based on ultrasound examination to provide the best treatment. The aim of this study was to describe the ultrasound features of ovarian masses detected during pregnancy and to optimize and personalize their management with the expertise of gynecologists, oncologists and sonographers. (2) Methods: Clinical, ultrasound, histological parameters and type of management (surveillance vs. surgery) were retrospectively retrieved. Patient management, perinatal outcomes and follow-up were also evaluated. (3) Results: according to the literature, these masses are most frequently benign, ultrasound follow-up is the best management, and obstetric outcomes are not considerably influenced by the presence of adnexal masses. (4) Conclusions: the management of patients with ovarian masses detected during pregnancy should be based on ultrasound examination, and a centralization in referral centers for ovarian masses should be considered.

## 1. Introduction

The incidence of adnexal tumors during pregnancy has increased significantly with the application of routine ultrasound to monitor pregnancy. Most adnexal tumors are discovered incidentally during the first trimester [1].

Although the management of adnexal masses in pregnancy is still controversial, in recent years, there has been an increase in clinical research on adnexal masses in pregnancy involving large study populations due to the increased diagnostic ability of experienced sonographers [2].

Adnexal cysts have been reported to be visible on ultrasonography in 4.1–24.9% of pregnant women [3,4]; however, the majority are benign and typically regress spontaneously or with expectant management; indeed, most ovarian tumors seen during pregnancy seem to disappear in the third trimester, in particular those smaller than 5 cm, at a rate of 71% to 89% [1,5,6]. However, adnexal masses may be challenging to categorize during pregnancy because hormonal changes related to the pregnancy could modify how ovarian masses appear. The most frequent adnexal masses in pregnancy are ovarian cysts, such as follicular cysts and corpus luteal cysts. Follicular cysts grow because of hormonal changes during pregnancy when a follicle that did not ovulate fails to regress on its own. In the early first trimester, progesterone is produced by the corpus luteum to sustain the pregnancy. By the eighth week of pregnancy, when the placenta takes over progesterone secretion, they often regress, but sometimes, they can persist and develop corpus luteal cysts.

Ovarian cysts identified in the early stages of pregnancy are ovarian endometriomas in about 4–5% of cases, while most adnexal cysts identified after 16 weeks of gestation are mature cystic teratomas. Decidualized endometriomas usually show large intraluminal papillary projections with increased blood flow that resemble malignant ovarian tumors. Papillary projections can make it difficult to distinguish between benign masses like decidualized endometriomas and cystadenofibromas and malignant ones like invasive and borderline tumors [7,8,9,10].

Malignant ovarian tumors (including those of low malignancy) account for about 1–8% of adnexal tumors in pregnancy [11]. If malignancy is suspected, treatment should be decided based on gestational age, stage of disease and patient preferences. In the early stages, surgery for ovarian cancer can be planned after 16 weeks of pregnancy and chemotherapy from the second trimester, as done for non-pregnant patients. In advanced disease, when complete cell reduction is not feasible, neoadjuvant chemotherapy can be used even during pregnancy. The outcome of patients with ovarian cancer diagnosed in pregnancy is similar to non-pregnant patients, and the stage of the disease is the most important prognostic factor [12].

Adnexal tumors are often asymptomatic and discovered incidentally. However, some of them are symptomatic due to the size, location or compression of adjacent structures. Complications such as torsion, hemorrhage, rupture and obstruction of labor can also occur [13]. The correct approach should be discussed in order to obtain the right balance between oncological risk, complications and maternal–fetal risks. Currently, ultrasound examination is considered as the first-line imaging method used to distinguish between benign and malignant ovarian tumors [14].

Magnetic resonance imaging (MRI) is useful when ultrasound is inconclusive, when there is a high risk of malignancy or when a more complete definition of histological aspects, such as their relationship to other organs, is needed. This allows for more precise tissue definition and more precise characterization of large masses that are not easily visualized by ultrasound. Recent data indicate that MRI exposure during pregnancy does not pose any risk to the mother or the newborn. On the contrary, there is no consensus regarding the use of gadolinium during pregnancy. Therefore, contrast-enhanced MRI should only be used when absolutely necessary [15].

The widespread use of ultrasound has enabled the accurate assessment of incidentally diagnosed ovarian lesions in asymptomatic pregnant women [1,4]. When a pelvic mass is suspected in a pregnant woman, transvaginal ultrasound is currently the first-line imaging method used to differentiate adnexal lesions. In cases of larger masses, transabdominal ultrasound is also used. On sonographic examination, the size and morphology of the lesions are the main sonographic features used to triage women with adnexal tumors for follow-up management or surgery. However, pattern recognition based on expert subjective assessment of adnexal tumor features remains the best method that can be used to preoperatively distinguish the type of ovarian mass in non-pregnant women [16].

For non-expert sonographers, other prognostic methods are available and include the GI-RADS system, International Ovarian Tumor Analysis (IOTA) group rules with simple descriptions (SDs), logistic regression models 1 and 2 (LR1 and LR2) and simple rules (SRs) [17]. The simple description method cannot be used in clinical practice in pregnant women, since one of the necessary conditions for determining the description of malignancy is an age greater than 50 years [16]. SRs use the presence or absence of specific and selective tumor features, such as the size, location and presence of solid parts, color Doppler blood flow and the presence of fluid in the pouch of Douglas. In non-pregnant patients, this ultrasonographic method used for the characterization of ovarian tumors has a specificity of 78% and a sensitivity of 87% [18]. However, there are important limitations to the use of the original SR scoring system. An adnexal lesion is considered benign when only B (benign) features are seen, and a lesion is classified as malignant when only M (malignant) features are seen on ultrasound. When adnexal tumors do not have B or M features or have both, the tumor should be considered unspecified [19]. However, other scoring systems developed by the IOTA team in recent years can also be used to classify the risk of malignancy in most adnexal tumors. These methods include the evaluation of different NEOplasias in the adneXa (ADNEX) model [20] introduced in 2014 and the simple rule risk (SRR) model proposed in 2016 [21].

The traditional treatment for ovarian tumors during pregnancy is surgery in the second trimester due to the risk of possible complications such as torsion, rupture and late diagnosis of malignancy. Recently, however, conservative management with US follow-up has been recommended due to the risks of surgery, including miscarriage, premature contractions and embolism. Surgery during pregnancy is indicated only if there are acute complications such as torsion, rupture or obstruction or if malignancy is suspected [2].

To our knowledge, studies generally emphasize the importance of a sonographer who can differentiate between benign and malignant adnexal masses; these allow clinicians to conduct the correct management and to prevent surgery in the first trimester, which involves anesthesia and a maternal–fetal risks. In our analysis, including the expertise of ultra-sonographers with more than 10 years of experience allowed us to change our daily management and predict the proper treatment.

The aim of this study was to describe the ultrasound features of ovarian masses detected during pregnancy and to optimize and highlight how the involvement of an expertise gynaecolostic oncologist sonographer leads us to the personalize and to tailor on patients the correct management.

## 2. Methods

### 2.1. Inclusion Criteria

This was a retrospective, single-center, cohort study performed at the Gynecology Unit, San Salvatore Hospital, L’Aquila, Italy, and approved by our Institutional Board (ID 01-2023). All patients had already provided written informed consent for their data to be collected and analyzed for scientific purposes.

We included in the study group pregnant women with an ultrasound diagnosis of adnexal mass during pregnancy referred to our center between January 2022 and July 2023. Exclusion criteria were ultrasound examination performed by a non-experienced sonographer, previously known ovarian malignancy and in vitro fertilization (IVF) pregnancies with ovarian cysts due to stimulation. We excluded from the study group patients who had not consented to this study.

### 2.2. Data Collection

Clinical and ultrasound features, histological assessment and management (follow-up vs. surgery) were retrospectively retrieved from the patients’ medical records. Gestational age at diagnosis, time of surgery, histology, management and follow-up were documented. Additionally observed were gestational age at delivery, type of delivery, indication of caesarean section, obstetrics and perinatal outcomes. In cases of bilateral adnexal masses, our analysis focused on the mass with the more complex ultrasonography shape.

Information about follow-up during and after pregnancy was reported, including the number of follow-up scans and the time intervals between scans. According to the literature, indications for surgery depended on suspicion of malignancy reported by the examiner according to pattern recognition, symptoms or prevention of complications such as torsion, rupture or obstacle to normal full-term pregnancy [17,22,23].

All clinical and ultrasound information was collected retrospectively and organized in an Excel database (Microsoft Office Excel 2017, Redmond, WA, USA). Results are shown as absolute frequency (%) for nominal variables and as median (range) for continuous variables.

### 2.3. Ultrasound Evaluation

Transvaginal ultrasound was used to examine all patients using a standardized technique; a transabdominal scan was added when necessary. The exams were performed with high-quality ultrasound equipment (Esaote Technos MP, Genova, Italy; Esaote MyLab 70 XVG, Genova, Italy; GE Voluson E8 Medical Systems, Zipf, Austria; Samsung Medison Hera9, Samsung Healthcare, Seoul, Korea) by a level III ultrasound examiner (M.L.) with more than 15 years of experience in gynecological ultrasound.

The following parameters were assessed: location and size of the lesion (three orthogonal diameters), unilateral or bilateral mass, type of mass (unilocular, unilocular—solid, multilocular, multilocular—solid, solid), presence of papillary projections (defined as any solid protrusion into a cyst cavity with a height ≥3 mm), number of papillary projections within the cyst, irregularity of the surface of papillary projections, presence of solid tissue different from papillary projections and presence of septa, ascites and/or fluid in the pouch of Douglas. Color content of the papillary projections and/or other solid tissue were subjectively estimated at power Doppler examination, using a color score (1 = no vascularization; 2 = minimal vascularization; 3 = moderate vascularization; 4 = strong vascularization). In cases of bilateral masses, the mass with the most complex ultrasound morphology was used. If the masses had similar morphology, the larger mass was used. All masses were described using the International Ovarian Tumor Analysis (IOTA) terminology [24]. The specific diagnosis suggested by the experienced ultrasound examiner in the original report was recorded.

According to the protocol outlined by Testa et al. in 2020 [25], the advice of clinicians was mostly based on morphological assessment of the adnexal mass at ultrasound. When a conservative approach with strict ultrasound surveillance was conducted, ultrasound was performed once a month until the term of pregnancy or till ovaries were not usually visible anymore. A post-partum evaluation when available was also reported.

When surgery was necessary, it was usually performed after the first trimester of pregnancy, during the caesarean section or after delivery. All surgical details were reported.

## 3. Results

We identified 17 patients with a diagnosis of ovarian mass detected during pregnancy. The clinical characteristics of the study population are shown in Table 1. The median age at diagnosis was 33 (range 23–39) years, and 15 patients were nulliparous (88.2%). The median gestational age at diagnosis was 10 (range 6–21) weeks. In five women (29.4%), a diagnosis of an ovarian mass had been made before pregnancy. At the time of data collection, 6 (35.3%) pregnancies were ongoing; pregnancy outcome was known for 11 women (64.7%); 9 delivered at term (81.8%), 6 (54.5%) vaginally and 5 (45.5%) by caesarean section and 2 (18.2%) had a preterm delivery. The median gestational age at delivery was 39 (range 27–41) weeks.

All women were managed expectantly, and no one required surgery during pregnancy due to the adnexal mass. Four women who attended a monthly scan had a reduction in cyst dimension compared to the initial measurement and complete resolution at a median of 26.5 weeks of gestation (range 14–35). Four (23.5%) patients underwent post-partum ultrasound follow-up, and four (23.5%) women underwent surgery. In two (50%) patients, the adnexal mass was removed during caesarean delivery. One patient had surgery during preterm caesarean section at 26 weeks because of fetal–maternal complications (preterm prelabor rupture of the membranes—pPROM and breech presentation); the histology report confirmed the benign nature of the lesion. Another patient had surgery during caesarean section due to myoma previa. The remaining two (50%) women underwent surgery at least 3 months after delivery. For the two patients undergoing surgery after pregnancy, a laparoscopic approach was used in both. No surgical complications were described. The histological diagnoses of the ovarian cysts are shown in Table 1. All (100%) lesions were benign, of which two (50%) were teratomas, one (25%) was serous cystoadenofibroma and one (25%) was a corpus luteum. The ultrasound characteristics of the masses are shown in Table 2.

Only one (5.9%) patient had bilateral masses. The median maximum diameter of the masses was 46 mm (range 18–121). In nine patients (52.9%), the masses were described as unilocular, in four (23.5%) as unilocular solid, in three (17.6%) as multilocular and in one (5.9%) as solid mass. Cysts with ground-glass echogenicity were observed in three (17.6%) cases, with mixed echogenicity in five (29.4%). The cyst content was anechoic in six (35.3%) patients, low level in one (5.9%) and hemorrhagic in one (5.9%). Papillary projections were observed in four (23.5%) patients. The median height of the largest papillary projection was 7.5 mm (range 3–13). The contour of the papillations was irregular in 1/4 (25%) of the cases, and papillation flow was present in 2/4 (50%) of the cases. The median maximum diameter of the largest solid component was 14 (range 8–38) mm. In color Doppler examination, the majority of the cysts (82.3%) had no vascularization. Acoustic shadows were observed in three cases (17.6%). No woman had fluid in the pouch of Douglas or ascites. Table 2 lists the diagnosis recommended by the ultrasound examiner in the original reports.

**Figure 1:**
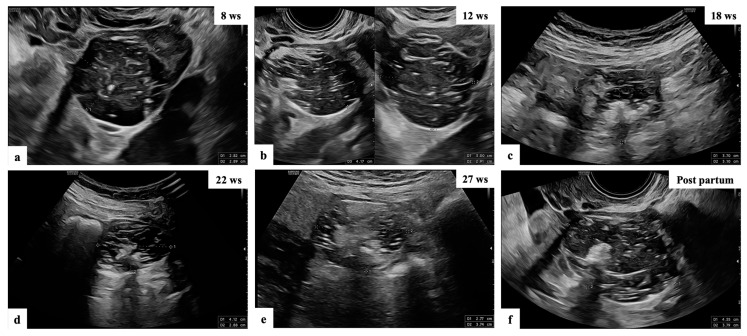
Ultrasound follow-up of patient n° 10 (**a**–**f**): grayscale ultrasound images showing adnexal masses with mixed content suspected of teratomas, as reported by the experienced examiner in the original ultrasound reports.

**Figure 2:**
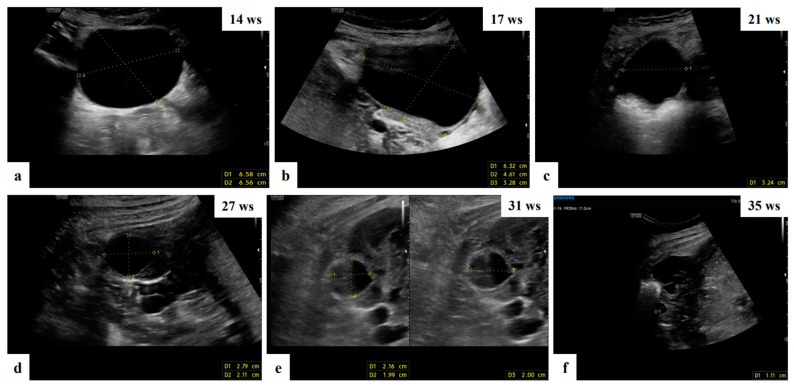
Grayscale ultrasound images showing adnexal anechoic unilocular mass suspected of functional cyst in ultrasound of 32 ys old patient managed expectantly during pregnancy who attended a monthly scan and had a reduction in the cyst dimension compared to the initial measurement and complete resolution at 35 weeks of gestation (**a**–**f**).

**Figure 3:**
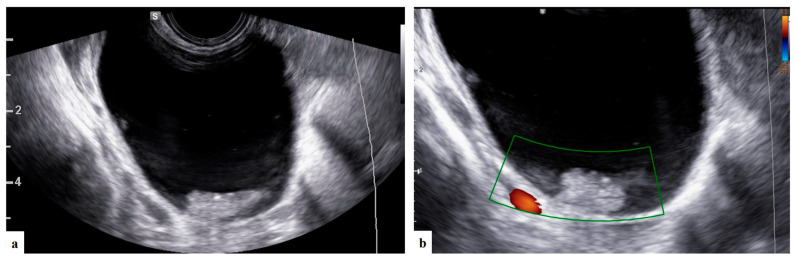
Grayscale and color Doppler ultrasound (**a**,**b**) images showing unilocular solid mass with anechoic content and papillary projection suspected of serous cystoadenofibroma (patient n° 5).

**Figure 4:**
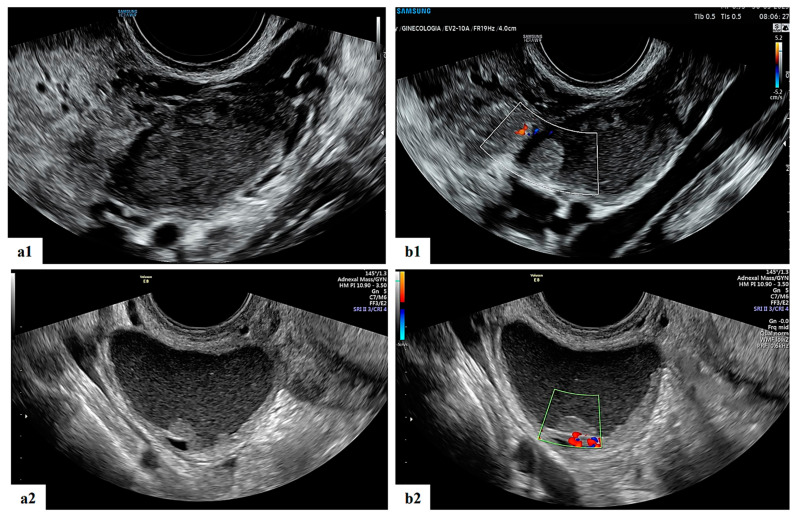
Grayscale (**a1**,**a2**) and color Doppler (**b1**,**b2**) ultrasound images of two decidualized endometriomas in our series (ongoing patients): moderately vascularized internal cyst wall, rounded papillary projections with smooth contours and cyst content with ground-glass echogenicity.

**Figure 5:**
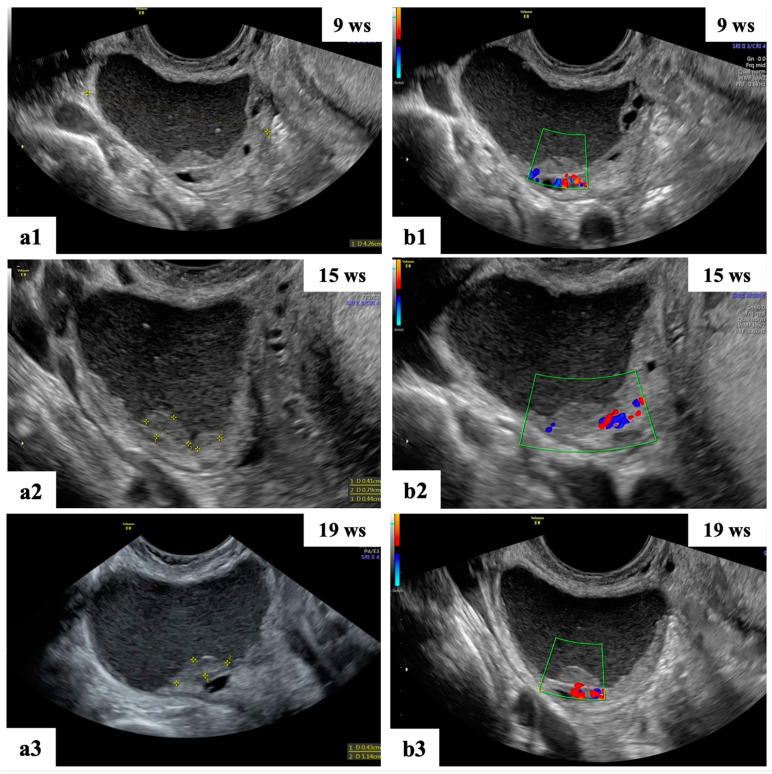
Ultrasound follow-up of the ongoing patient: grayscale (**a1**–**a3,b1**) ultrasound images showing unilocular mass with “ground glass” content suspected of decidualized endometrioma. The mass shows papillary projections with regular smooth contours and moderate vascularization at 15 wks (**a2**,**b2**) of gestation and reductions in the cyst dimension and number of papillary projections at 19 wks (**a3**,**b3**).

The operator diagnosed all 17 masses as benign. Five (29.4%) cases were teratomas (Figure 1 and Figure S1). Three (17.6%) cases were functional cysts (Figure 2), one (5.9%) case was cystoadenofibroma (Figure 3) and two (11.8%) cases were decidualized endometrioma (Figure 4 and Figure 5).

CA 125 results were available for 17 (100%) women and negative in all patients, with a median CA 125 concentration of 21 UI/mL.

Among the patients undergoing surgery, all four masses (100%) described as being most probably benign on ultrasound examination were confirmed based on final histology with correct specific diagnosis (two dermoid cyst and one serous cystoadenofibroma). One case misclassified as endometrioma was a corpus luteum based on final histology.

Obstetrics and perinatal outcomes were known for eleven patients and are shown in Table 3. All pregnancies were singleton. No patients had a miscarriage. Only two patients had preterm delivery. No newborns had an Apgar score < 7 at 1 min and 5 min and none died. Median fetal weight at delivery was 3280 (range 870–3525) gr. Median volume of blood loss during delivery was 500 (range 150–700) cc.

## 4. Discussion

The finding of adnexal masses in pregnancy is not frequent; the incidence varies from 0.04% to 1.3% of pregnancies [15]. Furthermore, the correct management has been recently investigated in the literature because the routine use of first-trimester ultrasound increased the diagnosis of adnexal masses in asymptomatic pregnant women [26].

The main goal of this study consists in determining the correct ultrasound instrumental diagnostics to allow for the implementation of the most correct management during gestation. Many ovarian ultrasound findings are functional cysts, including corpus lutea and follicular cysts. These represent about 30% of pregnancy masses and usually regress spontaneously during the first or early second trimester of gestation.

The percentage of borderline or oncological adnexal masses found is between 1 and 8% [27], and these are mainly due to related complications like torsion, hemorrhage, rupture and labor obstruction. These complications are common in all adnexal masses, although they are more severe in malignant ones [13].

The incidence of adnexal torsion during pregnancy is uncertain; estimates range from 0.2 to 3%, and it can occur at any time during gestation [28]. However, the main symptoms are nonspecific, and it is important to maintain a high level of suspicion of torsion in pregnant patients presenting with an adnexal mass and acute lower abdominal pain; a significant percentage (ranging from 38% to 60%) of pregnant patients with torsion may show normal Doppler flow on ultrasound examination [29]. In our specific case, the average size of the adnexal cysts (<5 cm) and the close clinical surveillance guarantee a timely diagnosis of this rare complication. Moreover, isolated fallopian tube torsion is an exceptionally uncommon occurrence in pregnant women, and its diagnosis may be difficult due to the presence of nonspecific clinical symptoms [30].

We emphasize that any hospital center can be faced with the management of these masses, even complex and rare cases; while clinicians generally concur on the treatment of complications arising from adnexal masses, the follow-up management of these lesions is subject to considerable debate, with different opinions on the best care plan.

Ultrasonography is the most commonly used tool for evaluating ovarian tumors during pregnancy because of its relative safety. The availability of a level III ultrasound center and a sonographer with more than 10 years of experience is a fundamental step in the daily practice of managing these issues.

The conventional first-line imaging method used for the assessment of adnexal disease is frequently recognized in clinical practice as a transvaginal ultrasound examination [31,32,33,34]. It has been demonstrated that the operator’s skill improves the accuracy of diagnosis via ultrasonography in discriminating between benign and malignant adnexal tumors [16,35,36]. This is crucial for both the diagnosis of adnexal lesions and the choice of management strategy.

In our series, we presented 17 pregnant women with an ultrasound diagnosis of adnexal mass during pregnancy from January 2022 and July 2023. We present these limited data numbers since recently we had the chance to have level III ultrasound scans carried out by an experienced sonographer.

The sonographer reported that all masses were benign; thus, unnecessary or ineffective surgeries could be avoided. Advanced ultrasound skills and the ability to differentiate benign from malignant in pregnancy allows for the number of surgeries to be reduced, the treatment to be personalized, and the appropriate management strategy to be chosen. These evaluations have also been expressed in other studies [2,25,37], and all agree on identifying the clinical characteristics of the patients and the ultrasound characteristics of the adnexal masses to allow for tailoring management.

For sonographers with different levels of experience, it may be difficult to distinguish between benign and malignant tumors detected in pregnant patients. If a woman is pregnant, some ovarian lesions may appear differently on sonography. However, most of the several histological adnexal entities in our investigation displayed predictable sonographic features.

For 36 pregnant patients with complex adnexal masses found during ultrasound examination, Czekierdowski et al. documented ultrasonography characteristics, therapy and outcomes [37]. Even when asymptomatic, persistent lesions with complex morphology identified during an ultrasound scan need to be carefully evaluated to rule out the possibility of malignancy.

IOTA, Sassone and Lerner are three ultrasonographic ovarian mass scoring systems that Lee et al. compared [2] and assessed as potential predictors of the risk of cancer in pregnant women. The maximum ovarian mass diameter, the maximum ovarian solid mass diameter, the inner wall structure, the wall thickness, the thickness of the septum and papillarity were the six ultrasonic characteristics that statistically significantly differed from one another.

Regarding management, during pregnancy, laparoscopic evaluation of the abdomen can be difficult, and, in this context, ultrasound could play an important role in tailoring management and personalizing the treatment for the patient. Centralization of these pregnant patients in a reference center for ovarian masses should be taken into consideration for the management of patients with ovarian masses discovered during pregnancy.

In 22 pregnant patients with ultrasound-detected malignant ovarian tumors, Moro et al. recently characterized the most significant characteristics of these tumors [38]. They also showed how uncommon ovarian cancer is, with only 22 malignant tumors found during a 17-year span. Malignant ovarian tumors found during pregnancy can be identified via ultrasound using similar morphological characteristics to those seen in patients who are not pregnant.

Furthermore, ultrasound and other tests are utilized to determine whether an otherwise asymptomatic adnexal mass should be handled expectantly or through surgical excision due to the high incidence of spontaneous remission. A thorough examination is crucial to determine the potential of complications including rupture, torsion and labor obstruction as well as to rule out the likelihood of cancer and probable benign masses.

In previous studies, no maternal or neonatal complications were reported for patients operated on because of borderline tumors or epithelial ovarian carcinomas [2,37].

Also, a recent investigation of 113 pregnant women with adnexal tumors by Testa et al. [25] showed that for patients in the surveillance group, as well as those with benign, borderline or primary epithelial invasive histology, no obstetric or neonatal problems were noted.

With regard to the incidence of malignancy, management and surveillance, our findings are consistent with those previously published in the literature [2,37]. Pregnancy significantly affects the biological behavior of ovarian endometriomas masses, according to our study. The majority of cysts are benign or decrease in size during pregnancy, with corpus luteal cysts, functional cysts and decidualized endometriomas showing the highest rates of regression (Figure 2 and Figure 5). Considering that we did not discover any malignant masses during our period of observation, the current study demonstrates that malignant ovarian masses in pregnancy represent a rare clinical disease.

The chance of distinguishing ovarian masses during pregnancy may help clinicians to decide the appropriate course of treatment for the patient. Making this distinction, for instance, would make it possible to avoid performing unneeded surgeries on adnexal masses during pregnancy. Expectant management appears to be a safe approach for functional cysts, which are likely to regress during pregnancy [39].

We highlight that in cases of small-size tumors, sub-specialization and competence in the field of gynecological oncology provide an excellent chance of an early diagnosis, which is necessary for obtaining personalized treatment, according to Di Legge et al. and Bruno et al. [40,41]. Furthermore, the patience of the expert sonographer represents a safe and reliable management for the patient. We therefore underline the importance of a diagnosis made by an expert sonographer of a benign mass, the most frequent in pregnancy, which most of the time does not require surgery.

Finally, we also confirm that the presence of ovarian masses in pregnancy does not invalidate the obstetric outcome of patients.

Our study’s strength is the availability of ultrasound photos or video clips for all cases of ovarian masses found during pregnancy that can be reviewed and eventually used to identify or confirm common imaging findings.

Indeed, the retrospective nature of the analysis, the low incidence of malignant ovarian masses in pregnancy and the single-center analysis are the main limitations. Furthermore, our small sample of patients represents a limitation for drawing definitive conclusions; more studies are needed to confirm these results prospectively. For example, comparison with MRI may also be useful in determining the optimal imaging technique, particularly for patients with adnexal masses who received an unreliable diagnosis from an US examination. We are currently conducting a study comparison between MRI and US-TV in pregnancy, and we are progressively enlarging the study population.

## 5. Conclusions

Ultrasound evaluation performed by an expert sonographer who can differentiate between benign and malignant adnexal masses is crucial because the management of adnexal tumors during pregnancy depends on the nature and type of the tumor and the complications that may arise. Conservative treatment is a good option for women with no symptoms and no signs of malignancy on imaging. In particular, our study showed that pregnant patients with ovarian masses should be centralized at a referral level III ultrasound center and that the treatment of these patients should be based on ultrasound examination performed by an expertized sonographer to permit the best management strategy to be chosen. To support our findings and outline a clinical care plan for pregnant patients with ovarian lesions, additional significant prospective trials are required.

## Figures and Tables

**Table 1:** Clinical characteristics of 17 women with ovarian cysts during pregnancy.

Characteristics	Value
Number of cases	17
Nulliparous	15 (88.2)
Ultrasound diagnosis before pregnancy	5 (29.4)
Age at diagnosis (years)	33 (23–39)
Gestational age at diagnosis (weeks)	10 (6–21)
Gestational age at last ultrasound examination (weeks)	26 (9–39)
Gestational age at delivery *	39 (27–41)
Delivery Spontaneous delivery Caesarean section Ongoing pregnancy	6 (35.3) 5 (29.4) 6 (35.3)
Management Follow-up Surgery after delivery Spontaneous resolution	9 (52.9) 4 (23.5) 4 (23.5)
Histological diagnosis ** Teratoma Serous cystoadenofibroma Corpus luteum	2 (50) 1 (25) 1 (25)

Data are given as median (range) or *n* (%); * information available for 11 patients; ** information available for 4 patients.

**Table 2:** Ultrasound characteristics of 17 women with ovarian cysts during pregnancy.

Ultrasound Characteristics	All (*n* = 17)
Bilateral mass	1
Maximum diameter of lesion (mm) (range)	46 (18–121)
Type of tumor Unilocular Unilocular solid Multilocular Multilocular solid Solid	9 4 3 0 1
Cyst content echogenicity Anechoic Low level Ground glass Mixed Hemorrhagic Not applicable (solid mass)	6 1 3 5 1 1
Color score 1 2 3 4	14 2 1 0
Maximum diameter of largest solid component (mm) (range)	14 (8–38)
Presence of papillary projections	4
Number of papillary projections 1 2 3	1/4 2/4 1/4
Papillation contour Irregular Smooth	1/4 3/4
Papillation flow Present Absent	2/4 2/4
Height of the largest papillary projection (mm) (range)	7.5 (3–13)
Presence of acoustic shadow	3
Presence of crescent sign	13
Diagnosis based on subjective assessment Benign Borderline Malignant	17 0 0
Specific diagnosis suggested by the original examiner Teratoma Functional cyst Decidualized endometrioma Paraovarian cyst Endometrioma Fibroma Cystoadenofibroma Sactosalpinx Corpus luteum	5 3 2 2 1 1 1 1 1

Data are given as median (range).

**Table 3:** Perinatal outcomes.

Patients	Gestational Age at Delivery	Singleton/Twin Pregnancy	Type of Delivery	Indication for Caesarean Section	Perineal Lacerations	Blood Loss during Delivery (cc)	APGAR Score	Fetal Weight (gr)
1	27	Singleton	Caesarean section and cystectomy	pPROM, breech presentation	-	500	7/8	870
2	40	Singleton	Vaginal delivery	-	Episiotomy and grade I laceration	700	8/9	3500
3	38	Singleton	Vaginal delivery	-	-	150	8/9	3450
4	41	Singleton	Caesarean delivery	Abnormal CTG	-	500	9/10	3185
5	38	Singleton	Vaginal delivery	-	grade I laceration	100	8/10	3070
6	39	Singleton	Caesarean delivery	-	grade II laceration	300	9/9	2880
7	40	Singleton	Vaginal delivery	Abnormal CTG	-	300	9/10	3525
8	40	Singleton	Caesarean delivery	-	Episiotomy	500	9/10	3300
9	38	Singleton	Vaginal delivery	Fetal malformation	-	500	7/9	3280
10	40	Singleton	Caesarean delivery	-	Episiotomy	200	9/10	3280
11	36	Singleton	Caesarean section and unilateral salpingo- oophorectomy	Myoma previa	-	600	8/9	2850

Information available for 11 patients.

## Data Availability

No new data were created or analyzed in this study. Data sharing is not applicable to this article.

## Supplementary Materials

The following supporting information can be downloaded at: https://www.mdpi.com/article/10.3390/diagnostics13203247/s1, Figure S1: grayscale ultrasound images of suspected teratomas.
