# Supplementary material for: Ultrasound Features and Clinical Outcome of Patients with Ovarian Masses Diagnosed during Pregnancy: Experience of Single Gynecological Ultrasound Center
Source: Diagnostics (Basel). 2023 Oct 18;13(20):3247. doi: 10.3390/diagnostics13203247 (PMC10606809; doi:10.3390/diagnostics13203247)
Supplement: Supplementary file 1 [file diagnostics-13-03247-s001.zip › diagnostics-2575335-supplementary.pdf]

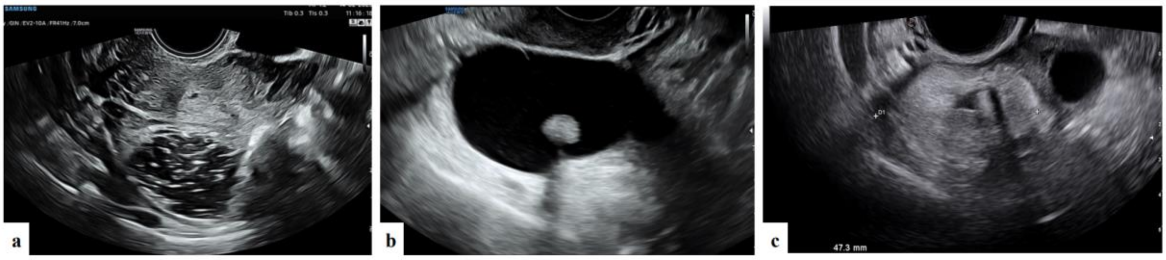

Figure S1. Grayscale ultrasound images showing adnexal masses suspected for teratomas: ongoing ultrasound follow up of three cases: (**a**–**c**). 'Dots and/or lines' (typical features according to the literature) were present in two cases masses (**a**,**b**). In the third case 'completely hyperechogenic lesion' (**c**).
